# Supplementary material for: Proteomic Analysis of Human iPSC-Derived Neural Stem Cells and Motor Neurons Identifies Proteasome Structural Alterations
Source: Cells. 2023 Dec 8;12(24):2800. doi: 10.3390/cells12242800 (PMC10742145; doi:10.3390/cells12242800)
Supplement: Supplementary file 1 [file cells-12-02800-s001.zip › cells-2670210-supplementary/Figure S3- Retroviral iPSC vs HFF beta subunits.pptx]

## Slide 1
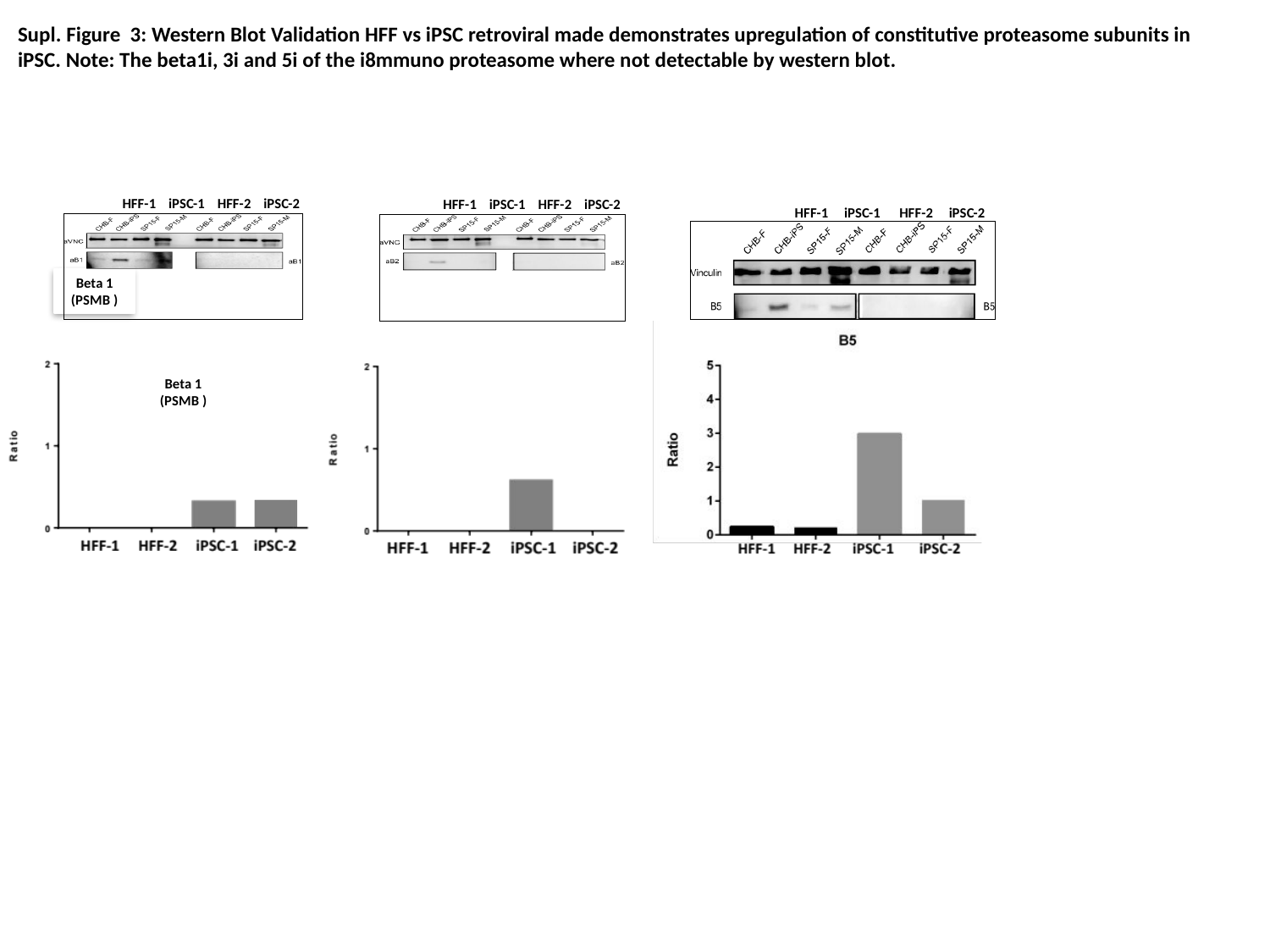

Supl. Figure 3: Western Blot Validation HFF vs iPSC retroviral made demonstrates upregulation of constitutive proteasome subunits in iPSC. Note: The beta1i, 3i and 5i of the i8mmuno proteasome where not detectable by western blot.
HFF-1 iPSC-1 HFF-2 iPSC-2
HFF-1 iPSC-1 HFF-2 iPSC-2
HFF-1 iPSC-1 HFF-2 iPSC-2
Beta 1
(PSMB )
Beta 1
(PSMB )
