# Supplementary material for: Proteomic Analysis of Human iPSC-Derived Neural Stem Cells and Motor Neurons Identifies Proteasome Structural Alterations
Source: Cells. 2023 Dec 8;12(24):2800. doi: 10.3390/cells12242800 (PMC10742145; doi:10.3390/cells12242800)
Supplement: Supplementary file 1 [file cells-12-02800-s001.zip › cells-2670210-supplementary/Figure S4.pptx]

## Slide 1
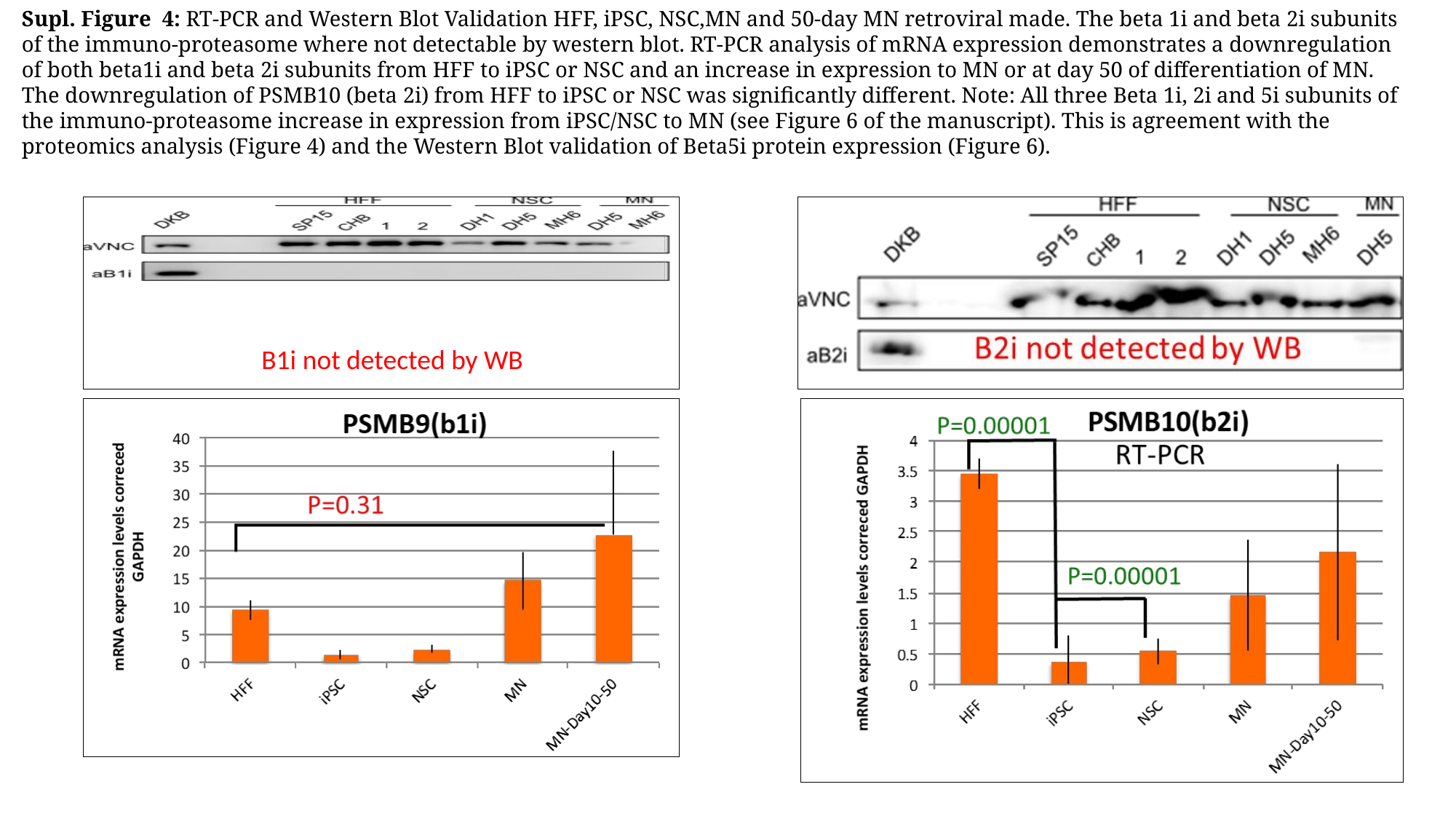

Supl. Figure 4: RT-PCR and Western Blot Validation HFF, iPSC, NSC,MN and 50-day MN retroviral made. The beta 1i and beta 2i subunits of the immuno-proteasome where not detectable by western blot. RT-PCR analysis of mRNA expression demonstrates a downregulation of both beta1i and beta 2i subunits from HFF to iPSC or NSC and an increase in expression to MN or at day 50 of differentiation of MN. The downregulation of PSMB10 (beta 2i) from HFF to iPSC or NSC was significantly different. Note: All three Beta 1i, 2i and 5i subunits of the immuno-proteasome increase in expression from iPSC/NSC to MN (see Figure 6 of the manuscript). This is agreement with the proteomics analysis (Figure 4) and the Western Blot validation of Beta5i protein expression (Figure 6).
B1i not detected by WB
