# Supplementary figures and images for: Proteomic Analysis of Human iPSC-Derived Neural Stem Cells and Motor Neurons Identifies Proteasome Structural Alterations
Source: Cells. 2023 Dec 8;12(24):2800. doi: 10.3390/cells12242800 (PMC10742145; doi:10.3390/cells12242800)

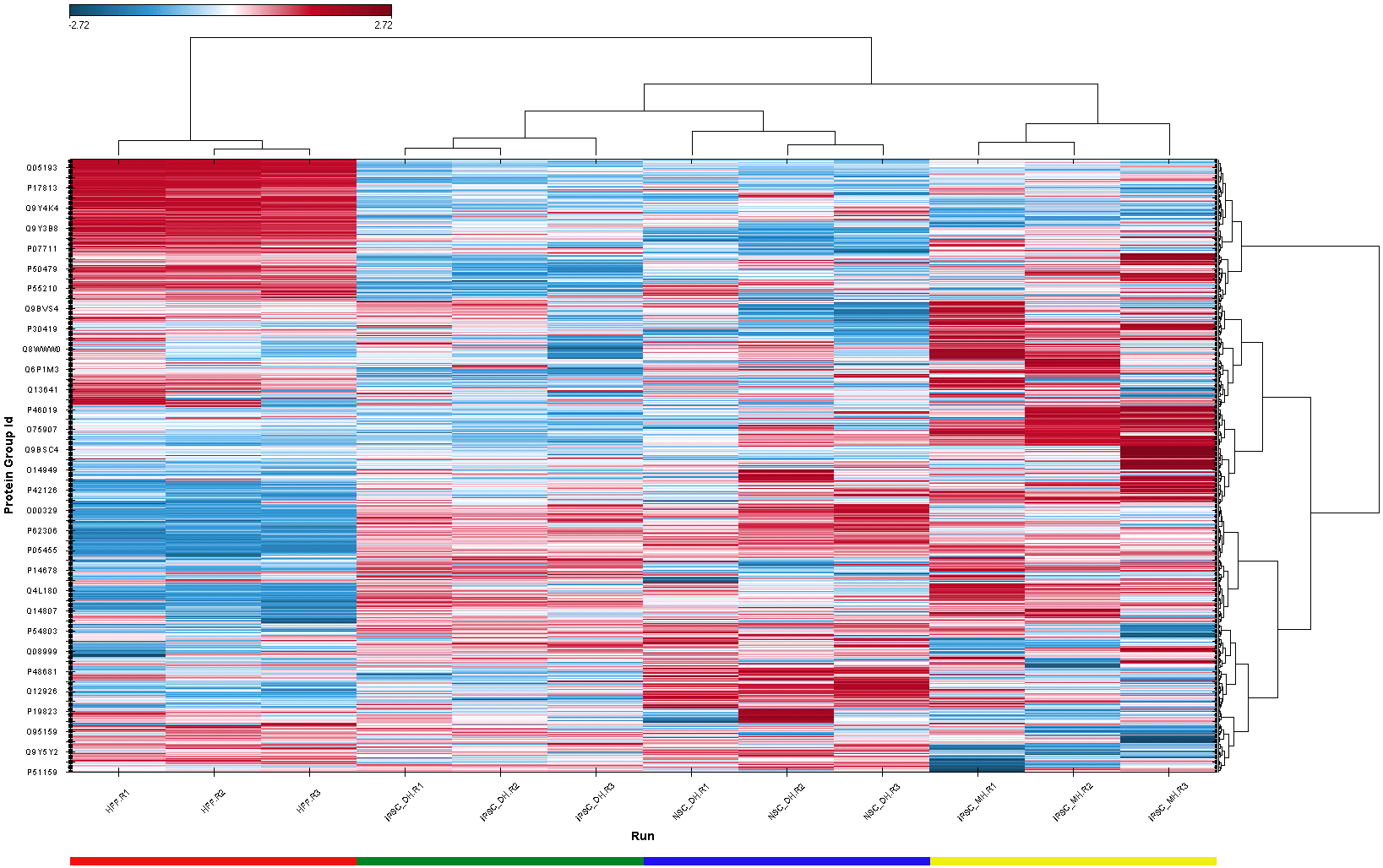

Supplement: Supplementary file 1 [file cells-12-02800-s001.zip › cells-2670210-supplementary/Figure S2 heat map.tiff]
